# Historical museum samples enable the examination of divergent and parallel evolution during invasion

**DOI:** 10.1111/mec.16353

**Published:** 2022-02-06

**Authors:** Katarina C. Stuart, William B. Sherwin, Jeremy J. Austin, Melissa Bateson, Marcel Eens, Matthew C. Brandley, Lee A. Rollins

**Affiliations:** ^1^ School of Biological, Earth and Environmental Sciences Evolution & Ecology Research Centre UNSW Sydney Sydney New South Wales Australia; ^2^ School of Biological Sciences Australian Centre for Ancient DNA (ACAD) University of Adelaide Adelaide South Australia Australia; ^3^ Biosciences Institute Newcastle University Newcastle upon Tyne UK; ^4^ 26660 Behavioural Ecology and Ecophysiology Group Department of Biology University of Antwerp Wilrijk Belgium; ^5^ 110067 Section of Amphibians and Reptiles Carnegie Museum of Natural History Pittsburgh Pennsylvania USA

**Keywords:** adaptation, divergent evolution, invasion, parallel evolution, *Sturnus vulgaris*

## Abstract

During the Anthropocene, Earth has experienced unprecedented habitat loss, native species decline and global climate change. Concurrently, greater globalization is facilitating species movement, increasing the likelihood of alien species establishment and propagation. There is a great need to understand what influences a species’ ability to persist or perish within a new or changing environment. Examining genes that may be associated with a species’ invasion success or persistence informs invasive species management, assists with native species preservation and sheds light on important evolutionary mechanisms that occur in novel environments. This approach can be aided by coupling spatial and temporal investigations of evolutionary processes. Here we use the common starling, *Sturnus vulgaris*, to identify parallel and divergent evolutionary change between contemporary native and invasive range samples and their common ancestral population. To do this, we use reduced‐representation sequencing of native samples collected recently in northwestern Europe and invasive samples from Australia, together with museum specimens sampled in the UK during the mid‐19th century. We found evidence of parallel selection on both continents, possibly resulting from common global selective forces such as exposure to pollutants. We also identified divergent selection in these populations, which might be related to adaptive changes in response to the novel environment encountered in the introduced Australian range. Interestingly, signatures of selection are equally as common within both invasive and native range contemporary samples. Our results demonstrate the value of including historical samples in genetic studies of invasion and highlight the ongoing and occasionally parallel role of adaptation in both native and invasive ranges.

## 1 INTRODUCTION

The ecological and economic impacts of invasive species are a growing concern in our globalized world. Increased intercontinental travel and trade is giving rise to new or reinforced invasion pathways (Turbelin et al., 2017), resulting in a great number of alien species becoming established and spreading within novel ranges (Hulme, 2009). The financial cost of invasive species within Australia is estimated to be in excess of $13 billion annually (Hoffmann & Broadhurst, 2016). With habitat clearing and climate change expected to favour invasive species over native ones, the environmental and financial costs of invasive species are only expected to rise in the future (Dukes & Mooney, 1999). Many studies of invasive species’ success involve examining evolutionary changes following introduction and focus on rapid adaptation to novel environments (Prentis et al., 2008). This information is vital for long‐term management of invasive populations.

Understanding evolutionary trends across a species’ native and invasive ranges will help determine important adaptive elements that aid species’ persistence in a changing world. Species that are invasive present a contrariety when they face population decline within their native range (Bishop, 2011; Delibes‐Mateos et al., 2009; Erfmeier & Bruelheide, 2010; Rogers et al., 2006). Research efforts should tackle ecological questions of conservation and invasion management concurrently, enabling us to understand how and why patterns of adaptation in a species’ native and invasive populations may differ. It is possible that the translocation and establishment process itself may select for traits that enable an individual to overcome otherwise detrimental environmental instability or other novel stressors, increasing general fitness (Callaway & Ridenour, 2004; Liu & Trumble, 2007). Understanding how the invasion process may induce differences in population persistence is made even more pressing by the increasing anthropogenic impact on the natural world, including ongoing land alteration, environmental contamination and human‐induced climate change (Hellmann et al., 2008).

Often, these adaptive changes are identified through contrasting present‐day native and invasive populations (Hofmeister, Stuart, et al., 2021). However, such approaches exclude the temporal element of species’ change, so that such studies assume native populations have not changed since the founders of the invasive population were collected. This would then lead to the conclusion that all similarities between native and invasive populations result from a common ancestral population and are not due to parallel change since separation. However, with global anthropogenic change impacting the natural world, it is reasonable to assume that altered or increased selection regimes have arisen during the post‐industrialized world, shaping species worldwide (Siepielski et al., 2017; Sokolova & Lannig, 2008). Historical specimens therefore provide an unparalleled tool to better contextualize divergent vs. parallel evolution, providing phenotypic and, more recently, genotypic information that can be used to identify temporal changes in species ranges and traits (Ewart et al., 2019; Lopez et al., 2020). Studies focusing on rapid local adaptation in invasive species may now make use of historical DNA alongside contemporary samples to understand the selective forces shaping both invasive and native ranges concurrently.

The common or European starling, *Sturnus vulgaris*, presents an ideal system to use historical samples to investigate both divergent and parallel genetic change within an invasive species. The European starling (hereafter starling) is a highly invasive pest, introduced and successfully establishing on every other continent except Antarctica (Higgins et al., 2006). Despite this, native range starlings are themselves a conservation focus, with declines of more than 50% in some countries (Versluijs et al., 2016) putatively associated with shifts in farming practice that are common in their native range (Freeman et al., 2007; Heldbjerg et al., 2016). Fortunately, due to the historical popularity of collecting bird skins, historical starling samples may be found scattered across many museums and institutions in both their native range and within invaded countries. These skins serve as untapped reserves of genetic information, which may be used to track temporal genetic changes across the native range, reveal information regarding historical population structure and provide context that enables us to better understand current patterns of native range starling decline.

**FIGURE 1:**
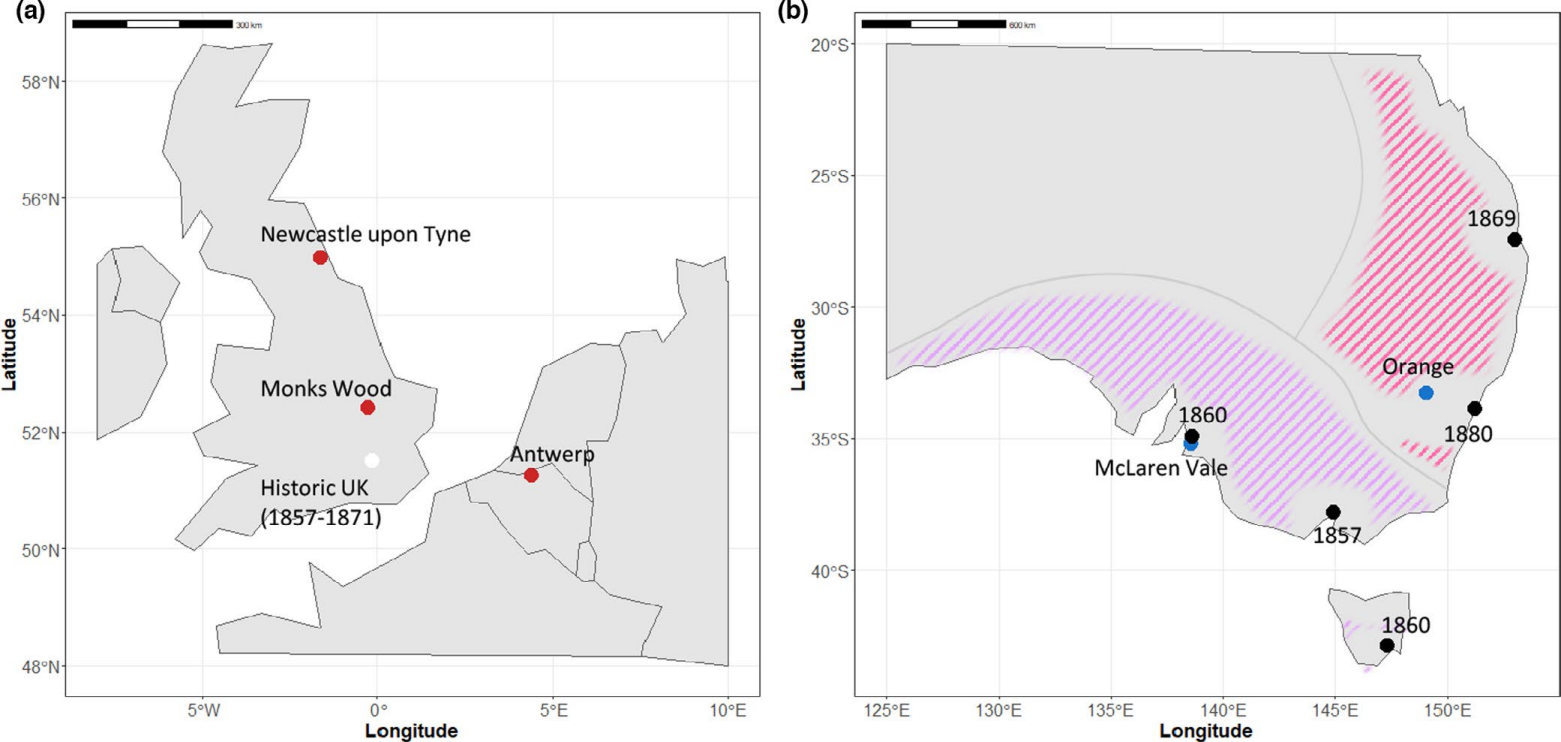
Geographical distribution of the *Sturnus vulgaris* collection sites in the United Kingdom and Belgium (native range, red points), Australia (invasive range, blue points) and historical samples (white point). The coloured shading on the Australian map denotes their Australian range, broken up into the two main subpopulations. Introduction sites are marked in black on the Australian map, with first introduction year listed adjacently

Starlings present a prime example of how the combination of data from invasive, native and historical populations can clarify our understanding of evolution in both native and invasive contexts. Introduced deliberately and repeatedly by acclimatization societies into several Australian coastal cities during the 1860–1980s (Figure 1), the starlings’ range now stretches across the continent's eastern and southern coasts (Long, 1981). Genetic analyses support strong population substructuring across the invasive Australian range (Rollins et al., 2009, 2011), with reduced‐representation sequencing data indicating the two main subpopulations probably resulted from allelic differences in founding populations at different introduction sites (Stuart, Cardilini, et al., 2021). The historical specimens available for this species were collected within 15 years of the earliest documented introductions to Australia in 1856 from the same native range location (around London, UK) (Long, 1981), providing a snapshot of native starling populations at the time when founders were transported to Australia.

To better understand patterns of population structure and signatures of selection present in the invasive Australian range, we used a reduced‐representation sequencing approach to compare contemporary Australian (AU) and native range (United Kingdom, UK; Belgium, BE) starlings to historical UK samples collected during the period when the Australian founders were collected. Moreover, this project explores proximate drivers of invasive species’ evolution in the face of novel selection provided by new environments. Specifically, we compare population structure of native and invasive contemporary starling samples, and we explore genomic divergence between contemporary and historical samples and assess the putatively adaptive capacity of these genomic changes. Finally, we use historical samples as a basis of comparison to determine genomic regions of parallel change in both the contemporary native and invasive populations to better understand global shifts in selective forces.

## 2 METHODS

### 2.1 Sample collection and extraction (historical starlings)

We sourced historical starling specimens (HS) from the Natural History Museum (NHM) in Tring, UK (*N* = 15). Historical samples were selected on the basis of sampling location (in the vicinity of London, thought to be where Australian founders were sourced; Jenkins, 1977), sample quality, completeness of the collection record and sample collection date (samples collected from 1857 to 1871, during the period when the Australian introductions took place; Higgins et al., 2006; Table S1), and specimen age (adult) (samples were of mixed sex).

DNA extractions of historical samples were conducted in a specialist ancient DNA laboratory at the Australian Centre for Ancient DNA, University of Adelaide. We rehydrated 3–4 mm^3^ dried tissue in 1 ml of 0.5 M EDTA for 2 h and extracted DNA using a Qiagen DNeasy Tissue Kit (Qiagen) as per the manufacturer's instructions. DNA was eluted twice with 40 µl of EB buffer (+0.05% Tween‐20) for a final elution volume of 80 µl.

### 2.2 Sample collection and extraction (contemporary starlings)

We sourced contemporary native range starling samples from two UK locations: Monks Wood (MW: *N* = 15, blood, ~100 km from London, where starlings were thought to be sourced for relocation to colonies such as Australia) and rural sites around the city of Newcastle upon Tyne (NC: *N* = 15, blood, ~300 km to the north of Monks Wood), and one Belgian (BE) location in Antwerp (AW: *N* = 15, blood, ~350 km to the southeast of Monks Wood) (Figure 1a). We sourced contemporary Australian samples from two locations, previously shown to represent two separate populations within the Australian invasive range (Stuart, Cardilini, et al., 2021): McLaren Vale in South Australia (MV: *N* = 15, blood) and Orange in New South Wales (OR: *N* = 15, muscle tissue) (Figure 1b). Contemporary DNA extractions were performed using the Qiagen Gentra Puregene Tissue kit as per the manufacturer's instructions.

### 2.3 Sequencing and genome variant calling

We sequenced 75 contemporary and 15 historical samples using the DArTseq protocol (Kilian et al., 2012), using a restriction enzyme double digest of *Pst*I–*Sph*I. The sequencing was conducted on a Hiseq 2500, producing 312,907,523 single‐end reads of raw data across the 90 samples (26,408,649 across the 10 successfully sequenced historical samples; Table S1).

We used the stacks version 2.2 (Rochette et al., 2019) pipeline (Rochette & Catchen, 2017) to process the DArTseq raw data. We used the *process_radtags* function to clean the tags, discarding reads of low quality (‐q), removing reads with uncalled bases (‐c) and rescuing barcodes and radtags (‐r). We used the Burrows–Wheeler aligner (bwa) version 0.7.15 (Li & Durbin, 2009) *aln* function to align the read data to the reference genome *S*. *vulgaris* vAU1.0 (Stuart, Edwards, et al., 2021). Using fastqc, we identified base sequence bias in the adapter region, and so the first five bases were trimmed (‐B 5) during alignment. The reads were then processed through bwa *samse* and samtools version 1.10 (Li et al., 2009), before single nucleotide polymorphism (SNP) variants were called through stacks *gstacks* (default parameters) and then *populations* (parameter information below).

We produced an unfiltered SNP data set by running stacks *populations* with no parameter thresholds specified. We used this data set to produce the unfiltered loci and site counts (Table S2), split the data into three separate files for further assessment of sequencing data (contemporary native range samples: MW, NC and AW; Australian samples: OR and MV; and historical samples: HS). We calculated variant base substitutions in vcfstats version 0.0.5 (Lindenbaum, 2015) and variant density mapped along the reference genome scaffolds using samtools *bedtools* function (window size 1,000,000 bp). We used the dartr version 1.1.11 (Gruber et al., 2018) function *glPlot* to create a smear plot of the mapped variant data across individuals and genomics sites. This resulted in a population genetic file of 239,538 SNPs. We also passed the raw single‐end read data through the stacks bwa‐mem, and the bowtie‐gatk variant calling pipeline (Appendix S1), to compare the quantity of site variant data that was successfully mapped and assess how these alternative variant calling approaches performed for reduced representation sequencing data sets that contained degraded historical DNA.

We generated a “population genetics” variant file by running stacks *populations*, filtering for a minimum per‐population site call rate of 50% (−r 0.5), a minimum populations per‐site of 2 (‐p 2) and a minimum loci log likelihood value of −15 (‐‐lnl_lim ‐15), with one random SNP per tag retained (‐‐write_random_snp). We used vcftools version 0.1.16 (Danecek et al., 2011) to filter the following parameters: maximum missingness per site of 10% (‐max‐missing 0.9), minor allele frequency of 2.5% (MAF; ‐‐maf 0.025), minimum loci depth of 2 (‐‐minDP 2), minimum genotype quality score of 15 (‐‐minGQ 15) and site Hardy–Weinberg equilibrium exact test minimum *p* value of .001 (‐‐hwe 0.001). We chose a high threshold for missingness to not bias the population genetics analysis against the historical samples, which had much higher levels of missingness than the contemporary samples. MAF filtering helps remove misreads, and HWE filtering removed highly non‐neutral loci, both of which are important for capturing neutral population substructure. After filtering, we calculated individual relatedness, and closely related individuals were removed so that there was only one representative from each cluster in the final data (Figure S1; five Monks Wood, five Newcastle and two Orange individuals removed). This resulted in a population genetic variant file of 3,840 SNPs used in the Section 2.4.

We generated a “selection” variant file by using stacks *populations* to align the raw reads for all samples (with ‐‐lnl_lim ‐15 ‐‐write_random_snp flags) and then used vcftools to filter out only SNPs present in at least 50% of the historical individuals (i.e., in at least five historical individuals), with additional quality filtering (‐‐minGQ 15 ‐‐minDP 2), resulting in 12,219 SNP sites. Only these sites were then retained to filter the original *populations* variant file, along with an MAF minimum of 2.5% to remove possible sequencing errors. This produced a data set that retained only SNPs sequenced in at least half the historical individuals, which would be necessary for selection analysis. We filtered the selection variant file to form five pairwise population SNP data comparisons: UK‐HS (UK populations MW, NC, BE and 10 historical individuals); AU_east_‐HS (AU population OR, and 10 historical individuals); AU_south_‐HS (AU population MV, and 10 historical individuals); UK‐AU_east_ (UK populations MW, NC, BE, and AU population OR); and UK‐AU_south_ (UK populations MW, NC, BE, and AU population MV). While the native range population may contain a mix of resident and migratory individuals, because we see minimal population structure in the native range and very small *F*_ST_ values (0.003–0.008) we decided to include all contemporary native range samples in this analysis. Conversely, population genetics data from this paper (see Section 3.1) and Stuart, Cardilini, et al. (2021) support the existence of distinct subpopulations within Australia due to historical demographic processes, and hence these subpopulations were treated as separate populations for this analysis (AU_east_ and AU_south_). Within each of these five variant file subsets, we retained SNP sites present in at least five contemporary individuals, because the file was already filtered for loci present in at least 50% (5/10) of historical individuals. This relatively lenient filtering was necessitated by the smaller number of genomic sample sites produced by the degraded DNA, and ensured minimal loss of the historical information that was present. This resulted in five pairwise population files used in the Section 2.5: UK‐HS (4,997 SNPs), AU_e_‐HS (4,900 SNPs), AU_s_‐HS (4,961 SNPs), UK‐AU_e_ (4,907 SNPs) and UK‐AU_s_ (4,942 SNPs).

### 2.4 Population structure analysis

We analysed the population genetics variant file in several ways to examine the population structure and differentiation across the native and invasive ranges, and between contemporary and historical native ranges. We used R version 3.5.3 (R Core Team, 2017) to run the snprelate *snpgdsPCA* function to create a principal components analysis (PCA) of the loci. We used admixture version 1.3.0 (Alexander et al., 2009) to determine individual ancestry proportions for each of the following three sample subsets: all samples, contemporary native range and historical, and contemporary Australian. We calculated marginal likelihood for model complexity (*K*, number of genetically distinct sources) 1–8 by averaging over 25 runs, and admixture proportion (Q) profiles were generated by clumpak (Kopelman et al., 2015) (run on default settings) to obtain an average Q profile. We used the stampp function *stamppFst* and *stamppNeisD* to calculate pairwise *F*_ST_ and Nei's genetic distance (Nei, 1972) between sampling locations. Finally, we used the dartr function *gl*.*tree*.*nj* to visualize the phylogeny of the six sampling groups (two contemporary Australian, three contemporary native range, and one historical sampling group).

### 2.5 Selection analysis

To obtain outlier SNPs putatively under selection between populations, the five pairwise selection analysis files were examined using two approaches.

When looking for diversifying selection, allele frequency‐based approaches are often used, such as bayescan version 2.1 (Foll & Gaggiotti, 2008). bayescan aims to identify SNPs subject to natural selection by assigning a per‐site posterior probability estimated by comparison of explanatory models with and without selection. We conducted bayescan SNP outlier analysis, with prior odds for the neutral model set to 10 (‐pr_odds 10), and a false discovery rate (FDR) of 0.05 (Figure S2).

Degraded historical DNA from older museum skins is known to have bias towards low‐diversity SNPs (Ewart et al., 2019), which may impact single site approaches to examining divergent selection. Therefore, we used an *F*_ST_ sliding window approach to identify genomic regions of putative diversifying selection. The vcftools *weir*‐*fst*‐*pop* function was used to analyse weighted *F*_ST_ in 900,000‐bp windows (10,000 window step). We chose this window size primarily based on the ratio of variants to genome size, with the chosen windows putatively spanning three to four variants, with small step sizes then allowing shifts in *F*_ST_ patterns to be pinpointed more exactly. We selected site windows that reported a weighted *F*_ST_ value in the top 99th percentile for each pairwise population selection analysis, and analysed the *F*_ST_ of SNPs within these windows in rank order. As putative outlier SNPs, we retained any SNP within these outlier windows that lay above an *F*_ST_ threshold relevant for each pairwise data set (this was determined visually as a plateauing of the ranked *F*_ST_ values, see Figure S3).

From the two above identification processes, we pooled putative outlier SNPs within each pairwise population comparison, to be used for further variant analysis.

### 2.6 Variant analysis and annotation

SNPs that were reported as outliers across either the UK‐HS or AU‐HS data set, as well as the UK‐AU data set, were designated as sites under divergent selection. SNPs that were reported as outlier across both UK‐HS and AU‐HS data sets (but not divergent between UK‐AU) were designated as sites under parallel selection. The remaining outlier SNPs we identified only in one data set: UK‐HS (putative UK selection), AU‐HS (putative AU selection) or UK‐AU (putative UK‐AU divergence). Classification of SNPs was conducted separately on the AU_east_ and AU_south_ Australian subpopulations, before being pooled. Further details are given in Section 3.3.

We analysed these five groups of SNPs for their functional roles and the nature of the mutation. We completed SNP analyses primarily using variant effect predictor (McLaren et al., 2016), using the genome annotation version released alongside the *S*. *vulgaris* vAU1.0 assembly (Stuart, Edwards, et al., 2021) to examine the functional consequences of the SNPs (processed to exclude multiple isoforms using agat *agat_sp_keep_longest_isoform*.*pl*; Dainat, 2020). We used bedtools and the agat function *agat_sp_functional_statistics* to extract genes and transcripts that overlapped the putative loci under selection, and extracted gene ontology (GO) terms. We used revigo (Supek et al., 2011) to visually summarize GO terms, and we calculated allele frequencies at SNP sites using bedtools.

Lastly, to test if there was an overrepresentation of SNPs located on the macrochromosomes (>20 Mb, as described in Backström et al., 2010), microchromosomes, or the Z sex chromosome, we used a Chi‐square test to examine the frequencies of these SNP types across four different SNP groupings: the divergent SNPs, the parallel SNPs, the remaining SNPs under selection, and the SNPs that were not flagged as being under selection in any of the pairwise data sets. We analysed these data using the *chisq*.*test()* function in R. To ensure results from this analysis were not artefacts of the data (due to diploid variant calling on hemizygous ZW females), we considered the sex of samples in our analysis. Because some individuals in this study were not sexed morphologically, we bioinformatically sexed individuals by assessing the inbreeding coefficient on the autosomes and Z sex chromosome, and conducted an analysis of variance (ANOVA) test to see whether major allele frequency was significantly associated with an interaction between SNP type (selection vs. nonselection) and SNP location (sex chromosome vs. autosome) using the *aov()* function in R. We constructed this analysis as many outlier identification methods employ allele frequencies for statistical comparisons between sample groups, and an interaction between SNP location and its categorization as under selection or not would be cause for concern about bias due to data artefacts.

## 3 RESULTS

### 3.1 Population structure of contemporary and historical *S. vulgaris*

**FIGURE 2:**
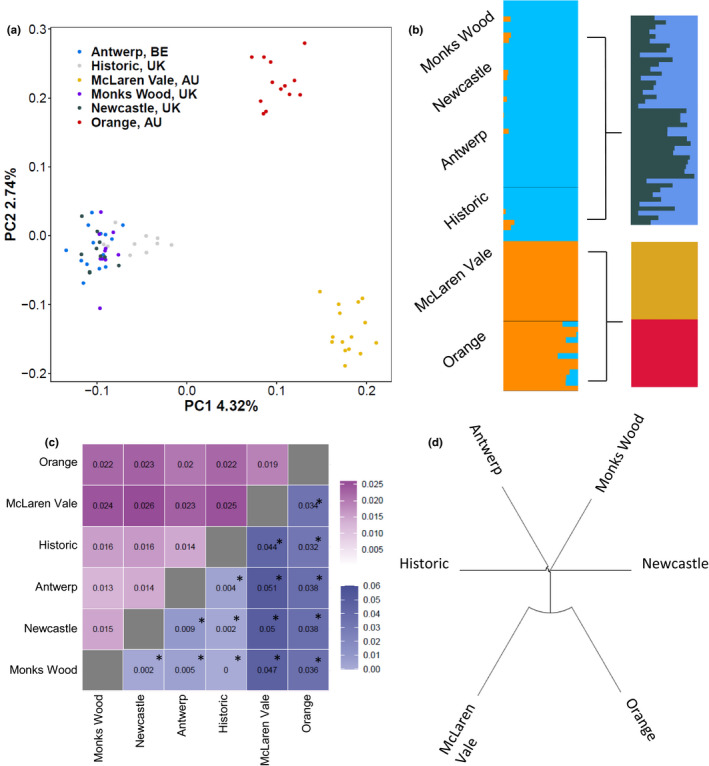
Population genetic analysis for contemporary and historical *Sturnus vulgaris* using the population genetics variant file. (a) PCA of the six sampling groups using snprelate, and (b) admixture ancestry Q profiles, averaged over 25 runs using clumpak for all sample groups, all native range samples (contemporary and historical), contemporary native range samples, and invasive Australian samples. (c) Heatmap of pairwise analysis between each of the sample groups, with above the diagonal (purple) Nei's genetic distance, and below the diagonal (blue) pairwise *F*_ST_ (an asterisk * denoting a significant *F*_ST_ result), and (d) the phylogenetic relationships between sampling sites using a neighbour‐joining tree

Our genetic data revealed strong differentiation between contemporary native and invasive range samples, as well as replicating the previously established subpopulation structure within Australia. PCA indicated that the historical samples were genetically clustered with contemporary UK populations, but were nevertheless the most similar native range population to the AU populations along the first PC axis (Figure 2a). Interestingly, the Australian samples appeared to be as divergent from each other as they were from native range samples (Figure 2a).

Through admixture analysis on the 73 sequenced samples (12 contemporary samples removed to due close relatedness, and five historical samples not included due to failed sequencing, see Section 3.4 “Sequencing and variant calling with historical samples”) we determined that *K* = 1 and *K* = 2 had similar support (Figure S4a). Ancestry proportions when plotted for *K* = 2 (Figure 2b) support the historical UK clustering with the contemporary native range, with very little further substructure revealed when admixture analysis was conducted just on the native range samples (Figure 2b; Figure S4b,c). Again, this analysis identified strong substructure in the Australian samples (Figure S4c).

Of the invasive Australian samples, we found that those from McLaren Vale showed greater genetic differentiation from the native range samples compared with those from Orange, evident in pairwise *F*_ST_ and genetic distance comparisons (Figure 2c) and corroborated by McLaren Vale admixture proportions lacking the UK cluster (Figure 2b). The historical samples were found to be most similar to samples from Monks Wood when considering pairwise *F*_ST_, PCA and admixture, whereas Antwerp appeared closest when considering genetic distance and phylogeny (Figure 2). We also characterized the relationship amongst the native and invasive range samples by the neighbourhood‐joining tree (Figure 2d), and found that the genetic differentiation between sampling sites across the native range was less than that across the invasive range.

### 3.2 Genomic divergence between contemporary and historical *S. vulgaris*

**FIGURE 3:**
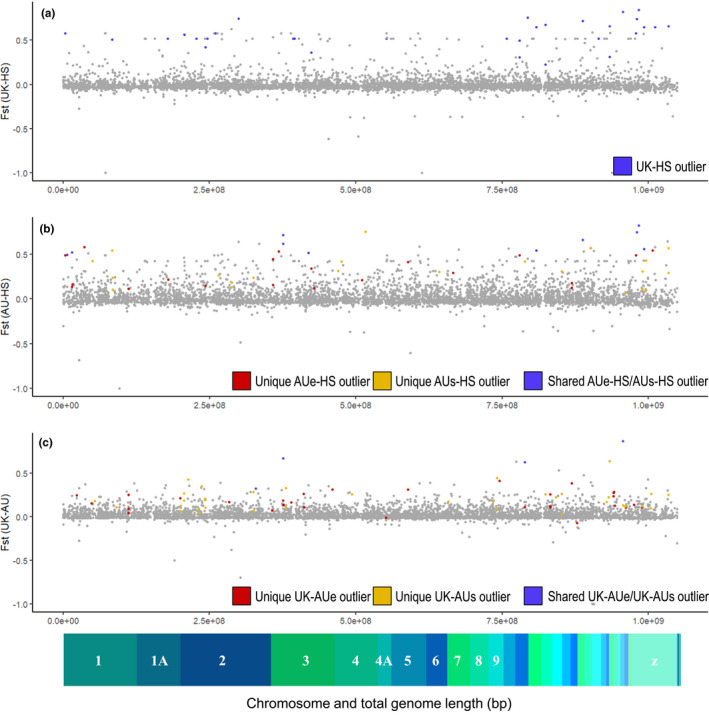
Pairwise population analysis of outlier loci across comparisons, mapped across the *Sturnus vulgaris* genome. Loci are plotted with respect to their position along the 1‐Gb genome with major scaffolds of the *S*. *vulgaris* genome assembly noted along the *x*‐axis, and with Weir and Cockerham *F*_ST_ calculated on a per‐loci basis using vcftools between pairwise populations on the *y*‐axis. (a) Flagged UK‐HS *F*_ST_ and outliers, (b) AU‐HS *F*_ST_ and outliers (red = unique AU_e_‐HS outliers, purple = unique AU_s_‐HS outliers, blue = shared AU_e_‐HS and AU_s_‐HS outliers), and (c) flagged UK‐AU (red = unique UK‐AU_e_ outliers, purple = unique UK‐AUs outliers, blue = shared UK‐AU_e_ and UK‐AU_s_ outliers). Nonoutlier SNPs are plotted in grey. The *x*‐axis depicts the major scaffolds of the *S*. *vulgaris* genome assembly, sized for their representation in the SNPs plotted in (a)–(c)

Using the default bayescan pipeline, we identified a total of 14 outlier loci across the five pairwise comparisons, eight of which were found in the UK‐HS comparison, four in the UK‐AU_e_ comparison, and one each in the AU_e_‐HS and UK‐AU_s_ comparisons (Table 1; Figure S2a–e). The sliding window approach indicated roughly similar numbers of outlier SNPs across all the population comparisons, with the highest number of putative outlier loci at 40 for UK‐AU_s_, and the lowest number at 27 for UK‐HS (Table 1; Figure S3). Interestingly, most but not all the SNPs identified by the bayescan FDR approach were also identified in the *F*_ST_ sliding window approach (Table S3). We visualized the outlier sites pooled across both outlier detection methods against the starling genome, revealing a uniform spread throughout the genome (Figure 3). In plotting the outlier SNPs specific to either the AU_east_ and AU_south_ populations, we found that while there was some overlap, a majority of flagged outlier SNPs were different across these two subpopulations. Furthermore, in superimposing these identified outlier SNPs on top of single SNP site *F*_ST_, we see that SNPs flagged as outliers within each population do not necessarily have high *F*_ST_ when the two populations are pooled and compared to either contemporary or historical native range samples.

**TABLE 1:** Number of putative sites under selection in *Sturnus vulgaris* reported by the different selective scans for the pairwise comparisons of the UK‐HS, AU_e_‐HS, AU_s_‐HS, UK‐AU_e_ and UK‐AU_s_ data sets

Outlier group		UK‐HS	AU_e_‐HS	AU_s_‐HS	UK‐AU_e_	UK‐AU_s_
Data set SNP count		4997	4900	4961	4907	4942
bayescan	FDR 0.05	8	1	0	4	1
*F*_ST_ sliding windows	Windowed *F*_ST_ >top 1% + SNP threshold	27	30	32	34	40
Total		31	30	32	35	40

We pooled putative outlier SNPs across the analyses (bayescan and *F*_ST_) done for each pairwise comparison, yielding a total of 31, 30, 32, 35 and 40 unique SNPs across identification methods for UK‐HS, AU_e_‐HS, AU_s_‐HS, UK‐AU_e_ and UK‐AU_s_ respectively (Table 1).

### 3.3 Putative adaptive selection

**FIGURE 4:**
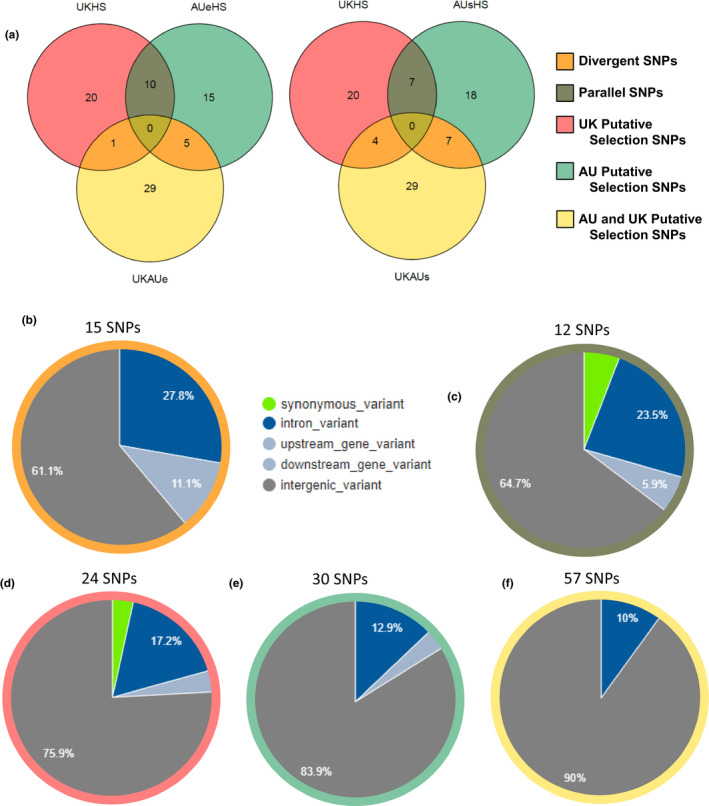
Summary of *Sturnus vulgaris* putative SNPs under selection. (a) Venn diagram of group categorization (left diagram using AU_east_ as the Australian comparison population, right diagram using AU_south_ as the Australian comparison population), and the remaining figures the variant effect predictor summary outputs of functional variation for each of the (pooled) five SNP groups of (b) SNPs under divergent selection, (c) SNPs under parallel selection, (d) UK SNPs under putative selection, (e) AU SNPs under putative selection, and (f) UK and AU SNPs under putative divergence

We identified SNPs under divergent and parallel selection, revealing a higher proportion of SNPs under parallel selection than divergent selection for AU_east_ comparisons (10 vs. six) and the reverse trend in comparisons involving AU_south_ (seven vs. 11; Figure 4a). We then pooled the results from the two Australian subpopulation analyses and, of the 5,068 tested SNPs, a total of 137 SNPs were identified as under selection (one SNP appeared in both the divergent and parallel SNP lists), of which 15 were identified as resulting from divergent selection, and 12 from parallel selection (Figure 4a, Table 2). Of these SNPs, one appeared as under divergent selection in comparisons to AU_south_ but under parallel selection when compared to AU_east_ (Table S4). The remaining SNPs that did not fulfil the criteria for divergent or parallel selection were categorized as resulting from putative UK selection (24), putative AU selection (30), and putative UK and AU divergent selection (57) (Table 2, Figure 4a).

**TABLE 2:** SNP and gene counts of *Sturnus vulgaris* SNPs putatively under selection, for the five categorical groups based on pairwise comparisons

Outlier SNP group	Gene identification	Divergent selection	Parallel selection	Putative UK selection	Putative AU selection	Putative UK & AU selection
AU_east_		6	10	20	15	29
AU_south_		11	7	20	18	29
Pooled		15	12	24	30	57
Coding regions	Total	8	6	16	14	40
	Known (blast+)	7	6	10	14	30
	Unknown	1	0	6	0	10

Across all five of these data sets, variant effect predictor analysis of the functional nature of the SNPs revealed that variants were mostly intergenic, and there were some within‐intron variants (Figure 4b–f). Of the SNPs under divergent and parallel selection, 70% were intergenic variants, with the remainder largely being made up of intron variants, and one synonymous variant being flagged as under parallel selection. SNPs identified as under UK and AU putative selection (either UK‐AU_e_ or UK‐AU_s_ divergence) contained the lowest proportion of intergenic variants (Figure 4f), and the only other SNPs with predicted protein coding sequences were present in the UK putative selection SNP list (Figure 4d).

While many of the SNPs mapped to unannotated loci, we mapped eight SNPs in the divergent data set to annotated genes, along with six SNPs in the parallel data set (Table 2; Tables S4 and S5). One SNP that was flagged in both the divergent and parallel data set mapped to the gene *Transforming Growth Factor Beta Receptor 2* (*TGFBR2*). Only one gene was found to be divergent within the native range (*Ankyrin repeat and KH domain*‐*containing protein 1*, *ANKHD1*), while four were found to be divergent within the invasive Australian range: *Glutamate ionotropic receptor kainate type subunit 2* (*GRIK2)*, *Neurexin*‐*3* (*NRXN3*), *Voltage*‐*dependent calcium channel subunit alpha*‐*2*/*delta*‐*3* (*CACNA2D3*), *Natterin*‐*4 (NATT4)* and *DNA methyltransferase 1* (*DNMT1*) (Table S5). A range of genes were flagged as being under parallel selection in both the native and invasive range: *E3 ubiquitin*/*ISG15 ligase TRIM25* (*TRIM25*), *Myb*‐*binding protein 1A*‐*like protein* (*MYBBP1A*), *CWC27 spliceosome associated cyclophilin* (*CWC27*), *Poly(A) RNA polymerase GLD2* (*TENT2*) and *Tripartite motif containing 28* (*TRIM28*) (Table S5). There was some gene ontology overlap between the two data sets (Figure S5).

**FIGURE 5:**
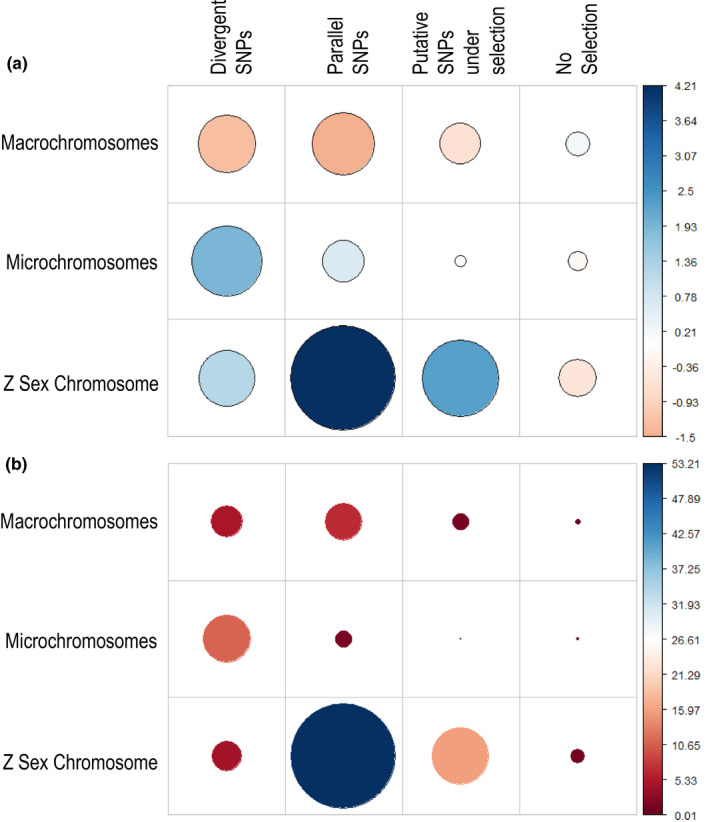
Test of statistical association between SNPs categorized as under selection vs. the chromosome type they reside in for *Sturnus vulgaris* DArT‐Seq, across SNP groupings of divergent SNPs, parallel SNPs, putative SNPs under selection (UK, AU, and UK and AU SNPs under putative selection), and SNPs not flagged as under selection. (a) Visualization of Pearson residuals, where the circle area is proportional to the amount of the cell contribution, positive residuals (indicating a positive correlation) are in blue, and negative residuals (indicating a negative correlation) are in orange; and (b) relative contribution of each cell to the total Chi‐square score

We tested for any bias in chromosomal location of loci under selection, using Chi‐squared analysis, and found that SNPs under putative selection were not proportionately distributed across macro‐, micro‐ and Z sex chromosome (*χ* ^2^_6_ = 33.23, *p* < .001, *N* = 5068). There was an overabundance of micro‐ and Z sex chromosome SNPs for all groups of SNPs under selection (divergent, parallel, and the UK, AU, and UK and AU SNPs under putative selection, i.e., those SNPs that were reported as an outlier in only one pairwise population comparison; Figure 5; Table S6). We conducted additional analyses on potential biases in major allele frequency, as well as bioinformatic sexing of individuals to determine if hemizygosity affected the results. We found no significant interaction between SNP location (sex chromosome or autosome) and SNP category (under selection or not) on major allele frequency (*F*_1,5064_ = 0.001, *p* = .98). Males and females were equally represented in our samples (Figure S6).

### 3.4 Sequencing and variant calling with historical samples

Of the 15 historical samples, 10 were successfully sequenced using DArTseq, a success rate (66.7%; Table S1) similar to that previously reported (62%) in a study using museum avian toe‐pad samples ranging from 5 to 123 years old (Ewart et al., 2019). We found no trends related to DNA concentration, sample age or fragmentation between historical tissue samples that were successfully sequenced vs. those which were not (Table S1; Figures S7 and S8).

Of the three variant calling pipelines (Table S2), we found that bowtie2‐gatk reported the highest percentage of successfully mapped reads over both contemporary and historical samples, followed closely by bwa‐mem. bwa‐aln resulted in much lower mapped read percentages. However, bowtie2‐gatk returned the smallest numbers of variant sites in the unfiltered and filtered data set, with bwa‐mem returning slightly higher values than bwa‐aln. These results are in agreement with previous assessments of these software performances for reads of approximately this length (Li, 2013; Li & Durbin, 2009). bwa‐aln is generally reported to map more conservatively than bwa‐mem (Robinson et al., 2017), leading to the much smaller mapped reads percentage, but this did not have a very large effect on the site counts or missing data per individual. The biggest difference between these two was the difference between the number of filtered variant sites for the historical samples, indicating that the lower quality reads produced by the historical samples were most impacted by the change in aligning algorithm. bwa‐aln was used as the variant calling pipeline in this paper because our read length fell on the border of what was recommended for bwa‐aln and bwa‐mem (70 bp), and a more conservative mapping and variant calling approach is suitable for population and selection analyses (when approaches are based on per‐site allele frequencies).

We assessed the unfiltered data, and the base substitution plots per population revealed that though the historical samples reported lower SNP counts, base substitution frequencies were similar across the three population groupings (Figure S9a–c). When we mapped and aligned these reads to the genome assembly alongside Illumina whole genome variant data for the species (Hofmeister, Stuart, et al., 2021), similar patterns were found between the two sequencing approaches, and across all three population groupings, although with lower resolution in the historical individuals (Figure S10). Our sequencing and mapping of the historical samples indicate that, despite the lower quality and fragmentation of DNA, the overall patterns of base substitutions resembled that of the higher quality fresh tissue used from the contemporary samples, and that the reduced representation approach reflected variant densities seen in whole genome sequencing analyses. A smear plot of data revealed that missing data are relatively evenly spaced along the genome for historical samples (Figure S11), and not centred on particular genomic regions or chromosomes. Finally, the MAF plots revealed slightly differing patterns of MAF across the six sample groupings sequenced but, importantly, the historical samples did not appear to contain an unusually high number of sites with very low MAF (Figure S12).

## 4 DISCUSSION

This study demonstrates that *Sturnus vulgaris* has not only undergone divergent selection within the invasive Australian range, but that the native and invasive ranges are undergoing parallel selection, possibly in response to global environmental changes. We note that contemporary native range populations, when considering comparative numbers of divergent SNPs, have undergone a similar amount of genetic change when compared to invasive range populations, despite the latter presumably being exposed to radically different and novel selection regimes. Moreover, we identified several genes related to immune function and pollution that appear to be under parallel selection in the contemporary native and invasive range samples, which may reflect global environmental changes over the last century and a half. The genes reported as divergent between the populations capture differing selection regimes driving evolution within the two contemporary populations. We also identified a bias for selection on the Z chromosome in comparison to the autosomes.

Importantly, this study has successfully used reduced representation sequencing of historical and contemporary specimens to examine selection in *S*. *vulgaris* within the native and Australian invasive ranges, demonstrating the utility of museum collections in aiding evolutionary studies. While the success rate and quality of the historical specimen sequencing reads was less than with their contemporary counterparts, the method nevertheless yielded sufficient SNP data to enable examination of population structure and description of temporal patterns of genomic change in starling populations. Museum resources remain largely untapped in genomic studies across a variety of biological systems, but may serve as an invaluable source of information that will significantly extend knowledge about evolutionary processes and change over time.

### 4.1 Population structure

Very few genetic data exist for native range *S*. *vulgaris*, and hence our study provides much needed insight into the population structure and genetic variation of the northwestern region of the native range. We identified low levels of genetic differentiation across the native range localities sampled. Some native range starlings are migratory (Feare, 1984), and this large‐scale dispersion undoubtedly helps to maintain genetic diversity and suppress local differentiation. As expected, the historical starlings bear a stronger genetic resemblance to contemporary samples from the native range than those from the invasive Australian population. The historical samples are most differentiated from their contemporary counterparts in the PCA as compared to analyses of admixture, *F*_ST_, genetic distance and phylogeny.

Records identify that the historical samples were taken from around London (Jenkins, 1977). The different population genetic analyses conducted indicated that either Monks Wood or Antwerp bears the strongest resemblance to the historical samples; these differences probably result from different statistical approaches underlying these analyses (e.g. *F*_ST_ considers the loci individually and ignores haplotypes, while PCA considers loci simultaneously and will include effects such as linkage disequilibrium between loci). The genetic differentiation between the two invasive Australian populations concurs with the two previously described Australian genetic subclusters (Stuart, Cardilini, et al., 2021), and further reinforces the idea that there were slight but distinct genetic differences in the founding individuals. Comparing the contemporary Australian sample sites to genotyping by sequencing (GBS) sequencing data from the same regions (Stuart, Cardilini, et al., 2021) suggests that the sample sizes in this study were sufficient to be representative of the genetic variation at sampling locations, and that between‐sample site genetic divergence is higher within this invasive population when compared to the native population over comparable geographical distance (Stuart, Cardilini, et al., 2021).

### 4.2 Genomic divergence

Only a few outliers were flagged by the bayescan approach, probably due to the small sample sizes giving low statistical power to this stricter analysis (i.e., the program was unable to pick up the low signals of selection in these recently diverged populations; Al‐Breiki et al., 2018). Nevertheless, pooling SNPs across both outlier methods enabled us to highlight a few key results regarding allele frequencies and outlier SNPs facing divergent or parallel selection across the starlings’ range.

Our results indicate that when assessing native and invasive genetic differences, supposed evolutionary divergence should not be attributed solely to novel invasive range selection pressures or the processes involved in invasion itself. Many studies utilize genetic comparisons between invasive and native ranges to examine evolutionary divergence (Leger & Rice, 2007; Liu et al., 2020; Querns et al., 2020). Research into evolution within invasive systems often focuses on the divergent evolution within the invasive range in response to invasion processes or new selection regimes (Lee, 2002). Despite the apparent conservatism over all loci (including neutral ones) in the native range, roughly equal numbers of SNPs were found to be under divergent and parallel selection, and native range divergence occurred at a similar rate to the number of diverging SNPs within the invasive range. It is apparent that neutral similarities or differences between sample groups (Figure 2, close genetic similarity between contemporary and historical native range) may not necessarily be indicative of the number of selective differences (Figures 3 and 4).

When examining the SNPs that were flagged as outliers against Australia‐wide calculated *F*_ST_ (Figure 3b,c), as well as subpopulation allele frequencies (Table S4), it is apparent that signatures of selection vary dramatically across the two subpopulations, particularly so for SNPs facing divergent selection. Divergence within the AU population may be a result of invasion bottleneck processes biasing allelic variation for or against rare variants (depending on their representation in the translocated individuals). However, given that several hundred individuals were introduced to Australia (Higgins et al., 2006; Jenkins, 1977), it would be very unlikely for common allelic variants to be lost through such random processes. A more likely explanation is that observed allele frequency shifts in AU are a result of factors that occurred after introduction, such as genetic drift, or selection against nonlocal maladaptation in which an invader's trait may be poorly matched to the new environment (e.g., Ward‐Fear et al., 2009). This result also emphasizes the importance of analysing selection separately for subpopulations within highly structured and demographically complicated invasions with multiple putative introduction sites, as is the case with many invasive populations (LaRue et al., 2011; Tay et al., 2020; Xia et al., 2020). In comparison, patterns of parallel selection are far more consistent across the two AU subpopulations, with similar frequency shifts in both AU_east_ and AU_south_ (Tables S4 and S5). In instances where the divergent allele frequency shifts have occurred in both AU subpopulations, this may indicate independent instances of selection, or alternately, as there is gene flow across the population, an allele could have reached high proportions in one subpopulation and spread to the other.

Parallel change across invasive and native ranges is difficult to detect without an outgroup population or historical samples, and thus has been little studied within a species’ invasive and native range. Here, we found that SNPs under putative parallel selection had higher allelic diversity in the historical samples, with both contemporary populations becoming fixed (or nearly so) for the same allelic variant (Table S4). This may be indicative of beneficial/nondeleterious alleles shifting towards fixation within contemporary populations, linkage with a nearby variant, or be a result of random processes such as drift (though this last explanation would be quite unlikely for parallel processes). This over‐representation of fixation in contemporary populations is unsurprising, because parallel evolution is more likely to have been based on standing genetic variation in the ancestral population (variants that were already there) than on the same novel variants arising independently in the two descendant populations. We do see apparent fixation in historical populations and more mixed allele frequencies within contemporary populations, in a small number of cases within the parallel SNP data.

Across all SNPs under selection, only a few resided in coding regions, with only a couple of these residing in protein coding regions. Other than intergenic variants, intron variants made up the highest proportion of SNPs. Despite not being transcribed gene regions, introns may function as gene regulatory regions, so polymorphisms may still elicit functional changes (Shaul, 2017), and even synonymous variants can show codon usage bias (Zeng & Bromberg, 2019).

Understanding broad genetic patterns behind rapid evolution has long been a focus of fundamental evolutionary biology. Here, we observed a bias towards the larger sex chromosome (Z) in terms of SNPs that were categorized as being under parallel selection (and also within the UK, AU, and UK and AU SNPs under putative selection). It is possible that this may have arisen due to biases in aspects of the data (e.g., sequencing method, SNP variant calling pipeline). However, we found no differences in major allele frequency across SNPs categorized as either under selection or not, across sex chromosomes and autosomes. The conclusion does align with theory that suggests that sex chromosomes are capable of playing a disproportionate role in evolutionary divergence due to their haploid nature in one sex (e.g., the “faster‐X effect”; Meisel & Connallon, 2013), and may be one of the first steps towards speciation (Oyler‐McCance et al., 2015; Wilson Sayres, 2018). The relationship between sex chromosome evolution and local adaptation has been demonstrated theoretically (Lasne et al., 2017) and experimentally (Lasne et al., 2019), and has previously been observed in the invasive *Drosophila suzukii* (Ometto et al., 2013). The latter finding, in conjunction with our results, suggests that rapid sex chromosome evolution may be a widespread phenomenon across many invasive taxa.

### 4.3 Genes undergoing putative adaptive selection

We identified a range of genes as under divergent or parallel selection in native and invasive range starlings, suggesting that a diverse range of biological processes are under selection across the species’ range. One gene, *TGFBR2*, was flagged as under parallel selection between UK and AU_east_, but divergent from AU_south_. *TGFBR2* has been well established as a top candidate gene for regulating differential beak morphology, and functional alterations in this gene are commonplace across many avian species (Abzhanov et al., 2006; Knief et al., 2012; Mallarino et al., 2011), suggesting a possible rapid response to changes in food source.

Examining genes under divergent selection across the starlings’ contemporary ranges allows us to consider possible drivers of selection that differ between populations. Of the other genes found to be under divergent selection in AU, three have been associated with cognitive function and learning in birds, and may be linked to cognitive selection processes during urban colonization. First, *GRIK2* may be involved in the functional molecular organization of the avian cerebrum (Jarvis et al., 2013), *CACNA2D3* is involved in neurexin‐mediated retrograde signalling (Tong et al., 2017) and may play an important role in pathways for learned avian vocalization (Friedrich et al., 2019; Wada et al., 2004), and finally *NRXN3* is related to brain connectivity (Mueller et al., 2020). *DNMT1* plays a central role in epigenetic inheritance because it copies methylation patterns following replication (Goyal et al., 2006), and may play an important role in rapid heritable responses within an invasion (Marin et al., 2020). In some cases, it is not easy to see how the function relates to possible drivers of selection; for example, *ANKHD1* was the only annotated gene we identified that appears to be undergoing divergent selection within the native range. This gene plays an important role in cell cycle progression and proliferation, and has been associated with cancers in humans and model organisms (Dhyani et al., 2012; Machado‐Neto et al., 2014). Although the biological functions associated with these genes are broad, this result provides candidate genes for future studies investigating epigenetic inheritance and cognition, and their relationship to invasion success within this and other species.

There are few studies that focus on parallel selection between invasive and native ranges within the same species, with research often focused on parallel changes across independent introductions to better understand invasion mechanisms (Popovic et al., 2021; Stern & Lee, 2020). Here we examined signatures of parallel selection to better understand the types of selection pressures on a globally widespread species. The gene *MYBBP1A*, also putatively under parallel selection in our study, has previously been found to be up‐regulated in response to pollution stress (Kumazawa et al., 2015; Mitra et al., 2020). Over the last 160 years, increased modernization globally has resulted in organisms being exposed to increased levels of pollutants and other immune system triggers (Capilla‐Lasheras et al., 2017; Cummings et al., 2020; Watson et al., 2017). Even trace amounts of compounds may be detrimental to some organisms (Bucci et al., 2020; Kozlov et al., 2009), and starling eggs have been demonstrated to accumulate polluting organic compounds (Eens et al., 2013). There are less obvious putative links to potential global drivers of selection for other identified parallel genes *CWC27*, which is tentatively related to inflammation and retinal degeneration (Busetto et al., 2020), and *TENT2*, possibly associated with post‐transcriptional gene regulation and epitranscriptomics (Menezes et al., 2018). Of the genes that have possibly undergone parallel selection in both the native and invasive range, we identified two from the tripartite motif protein family (*TRIM25* and *TRIM28*), which is involved in pathogen recognition and host defence pathways in numerous avian species (Blaine, 2013; Wei et al., 2016). Nevertheless, with global bird numbers declining across both rare and common species (Gross, 2015; Li et al., 2020), understanding selective regimes may assist our understanding of species and range persistence patterns, and may shed light onto the various factors influencing native range starling declines.

The genes discussed above represent a short and analytically conservative list of those putatively under selection across the starling's global ranges. There are undoubtedly more genomic sites under selection, either only identified in one of the pairwise outlier data sets (and so not categorized as parallel or divergent), or in linkage with the identified SNPs (Brodie et al., 2016), or not sequenced at all using this reduced representation approach. However, these variants and genes serve as a shortlist of suitable targets for future gene expression studies, or as the basis of the development of further hypotheses regarding selection regimes of global avian populations.

### 4.4 Historical sample sequencing

The success rate of historical sample sequencing was ~70%, and we found no correlation between sample properties and sequencing success. Patterns of variant density across the genome identified using DArTseq data appeared to follow similar patterns to the high‐quality variant density data set provided by the whole genome comparison (Figure S10), although historical samples did result in a patchier variant distribution. However, prior simulations using data from historical samples indicated that though historical samples contain significantly more missing data when compared to fresh tissue samples, the level of genotyping error had a minimal effect on population structure inference (Ewart et al., 2019).

In sequence data processing, the bwa aln‐stacks and bwa mem‐stacks pipelines performed similarly, with bowtie‐gatk performing comparatively much worse when considering total called SNPs. This large difference may be due to the large amounts of missing data (Catchen et al., 2013), but is different from the relative performance previously reported for stacks and gatk variant calling pipelines (Wright et al., 2019), suggesting that variant calling success is data set‐specific. Lastly, while processing of historical samples on their own resulted in lower levels of missingness, the comparatively lower number of called SNPs means that processing historical samples alongside contemporary counterparts resulted in a larger number of called variant sites.

### 4.5 Future directions

Greater geographical coverage of native range starling genetics is a vital future step for evolutionary genomic studies on this species. Improved native range genetic data will help us better understand population structure and allelic shifts in the invasive ranges, and also shed light on native range dispersal dynamics. Further, the success of the museum sample sequencing demonstrated here gives hope that when future large‐scale genomic studies are conducted in the native range, historical samples will be able to provide crucial background information regarding genetic diversity prior to the significant contemporary population declines.

Analyses of a greater number of historical samples will also aid in the categorization of adaptive SNPs, because this will reduce possible effects of random sampling bias and capture more rare alleles. The sequencing failure rate of this study is comparable to another study using similarly aged museum skins (Ewart et al., 2019), suggesting that future projects seeking to use museum samples might expect similar failure rates (30%–40%) and adjust their sampling design accordingly. Finally, similar analyses may be conducted between the historical UK and the well‐studied contemporary North American starlings (because this population has a similar introduction time, range size and environmental variation: Bodt et al., 2020; Hofmeister, Werner, et al., 2021). Further studies into the temporal changes this species has undergone may be extended upon through comparisons with other invasive populations in New Zealand, South Africa and South America. Comparisons across parallel introductions will provide an invaluable opportunity to contrast concurrent species invasion and selection across multiple different environments, allowing for the discovery of broad evolutionary patterns in this invasive species.

## 5 CONCLUSIONS

Overall, this study demonstrates that the combination of native, invasive and historical genetic data can lead to a more thorough understanding of global species shifts and adaptation during the Anthropocene. We used genetic sequencing of museum specimens to identify putatively adaptive genetic changes through reduced representation sequencing and outlier SNP identification analyses. We have described evidence of parallel and divergent evolution in native and invasive starlings since the mid‐19th century. Finally, we identify an apparent bias towards putatively adaptive SNPs on the Z chromosome, suggesting that the major sex chromosome may play a major role in rapid evolution within this species.

## AUTHOR CONTRIBUTIONS

Project conception: K.C.S., L.A.R. Sample collection: M.B., M.E., M.C.B., L.A.R. Laboratory work: K.C.S., J.J.A. Data analysis: K.C.S. Manuscript writing: K.C.S. Manuscript Editing: all authors.

### OPEN RESEARCH BADGES

This article has earned an Open Data Badge for making publicly available the digitally‐shareable data necessary to reproduce the reported results. The data is available at (https://www.ncbi.nlm.nih.gov/bioproject/).

## Supporting information

Supplementary Material

## Data Availability

The raw sequencing data have been deposited under BioProject accession no. PRJNA781785 in the NCBI BioProject database (https://www.ncbi.nlm.nih.gov/bioproject/) (Stuart, Sherwin, et al., 2021). Processed genetic data files and basic code is available on Dryad (https://doi.org/10.5061/dryad.dbrv15f2v) and Zenodo (https://doi.org/10.5281/zenodo.5839093). More fully annotated and cleaned code, along with some project vignettes and any other relevant files or metadata for this project are available on GitHub (https://github.com/katarinastuart/Sv4_HistoricalStarlings).
