# Supplementary material for: Historical museum samples enable the examination of divergent and parallel evolution during invasion
Source: Mol Ecol. 2022 Feb 6;31(6):1836–52. doi: 10.1111/mec.16353 (PMC9305591; doi:10.1111/mec.16353)
Supplement: Supplementary file 1 — Supplementary Material [file MEC-31-1836-s001.docx]

**Supplemental Information for:**

**Historical museum samples enable the examination of divergent and parallel evolution during invasion**

Katarina C. Stuart1, William B. Sherwin1, Jeremy J. Austin2, Melissa Bateson3, Marcel Eens4, Matthew C. Brandley5,6, Lee A. Rollins1

1 Evolution & Ecology Research Centre, School of Biological, Earth and Environmental Sciences, UNSW Sydney, Sydney, New South Wales, Australia

2 Australian Centre for Ancient DNA (ACAD), School of Biological Sciences, University of Adelaide, Adelaide, SA, Australia

3 Institute of Neuroscience, Newcastle University, Newcastle upon Tyne, UK

4 Department of Biology, Behavioural Ecology and Ecophysiology Group, University of Antwerp, 2610 Wilrijk, Belgium

5 Section of Amphibians and Reptiles, Carnegie Museum of Natural History, Pittsburgh, PA, USA.

6 Powdermill Nature Reserve, Carnegie Museum of Natural History, Rector, PA, USA.

Corresponding Author: Katarina Stuart ([Katarina.Stuart@unsw.edu.au](mailto:Katarina.Stuart@unsw.edu.au))

**Table of Contents:**

| **Appendix 1:** Alternate variant calling pipelines | Page 3 |
| --- | --- |
| **Figure S1:** Relatedness of *Sturnus vulgaris* contemporary and historical  samples | Page 4 |
| **Figure S2:** *Sturnus vulgaris* outlier analysis BAYESCAN plots | Page 5 |
| **Figure S3:** *Sturnus vulgaris* outlier analysis SNP FST plots | Page 6 |
| **Figure S4:** *Sturnus vulgaris* admixture cross validation (CV) error profiles | Page 7 |
| **Figure S5:** REVIGO output of biological processes GO terms associated with  putative genes under selection in *Sturnus vulgaris* | Page 8 |
| **Figure S6:** Bioinformatics sexing of *Sturnus* *vulgaris* | Page 9 |
| **Figure S7:** Gel of historical *Sturnus* *vulgaris* samples | Page 10 |
| **Figure S8:** Historical *Sturnus* *vulgaris* sample sequencing assessment | Page 11 |
| **Figure S9:** *Sturnus* *vulgaris* SNP data per base substitution counts | Page 12 |
| **Figure S10:** *Sturnus* *vulgaris* WGS and reduced representation sequencing SNP  variant density | Page 13 |
| **Figure S11:** Smear plot of *Sturnus* *vulgaris* reduced representation  sequencing SNP data | Page 14 |
| **Figure S12:** *Sturnus* *vulgaris* reduced representation sequencing SNP data  MAF profiles | Page 15 |
| **Table S1:** *Sturnus* *vulgaris* historical native range museum samples  (Tring BMNH) metadata | Page 16 |
| **Table S2:** Mapping reads of historical and contemporary *Sturnus* *vulgaris* | Page 17 |
| **Table S3:** *Sturnus* *vulgaris* SNPs discovered by each outlier identification  method | Page 18 |
| **Table S4:** Summary of allelic frequency for outlier SNPs in *Sturnus* *vulgaris* | Page 19 |
| **Table S5:** Summary of putative genes under spatial and temporal  selection in *Sturnus* *vulgaris* | Page 20 |
| **Table S6:** Chi-square table for the test of statistical association between  SNPs categorised as under selection in *Sturnus vulgaris* versus the chromosome type | Page 22 |

**Appendix 1: Alternate variant calling pipelines**

In addition to the bwa *aln* pipeline, the bwa *mem* and gatk variant calling pipeline was run on the cleaned and processed raw data produced by *process_radtags*. bwa *mem* was run on default parameters, before being processed by stacks *gstacks* and *populations*. For the gatk pipeline, bowtie2 was used for alignment (--phred33 --very-sensitive-local –I), Samtools to produce a sorted bam file. The Picard v2.18.26 (under Java v8u121) *BuildBamIndex* function was used to index the reads. The gatk *HaplotypeCaller* function was used to call SNPs and assemble the haplotypes separately for eachs ample. The gatk functions *CombineGVCFs* and *GenotypeGVCFs* were used to combine each individual gvcf file into one vcf file for all individuals

For comparison to the primary variant data set, two filtering parameters were used. For bwa *mem*, no filtering parameters were used in *populations*, and a filtered file was produced using the exact same filtering parameters as for the primary variant calling pipeline pipeline (Stacks *populations*: -r 0.5 -p 2 --lnl_lim -15 --write_random_snp, and VCFtools --max-missing 0.85 --maf 0.025 --minDP 5 --minGQ 15). For gatk, as the stacks *population* function is not used, the filtering parameters could not be replicated exact, instead the vcftools parameters --max-missing 0.5 --maf 0.025 were used.

**REFERENCES:**

Stelzer G, Rosen N, Plaschkes I, Zimmerman S, Twik M, Fishilevich S, Stein TI, Nudel R, Lieder I, Mazor Y *et al.* 2016 The GeneCards Suite: From Gene Data Mining to Disease Genome Sequence Analyses. *Current Protocols in Bioinformatics* **54** 1.30.1-1.30.33. (doi:10.1002/cpbi.5)

**
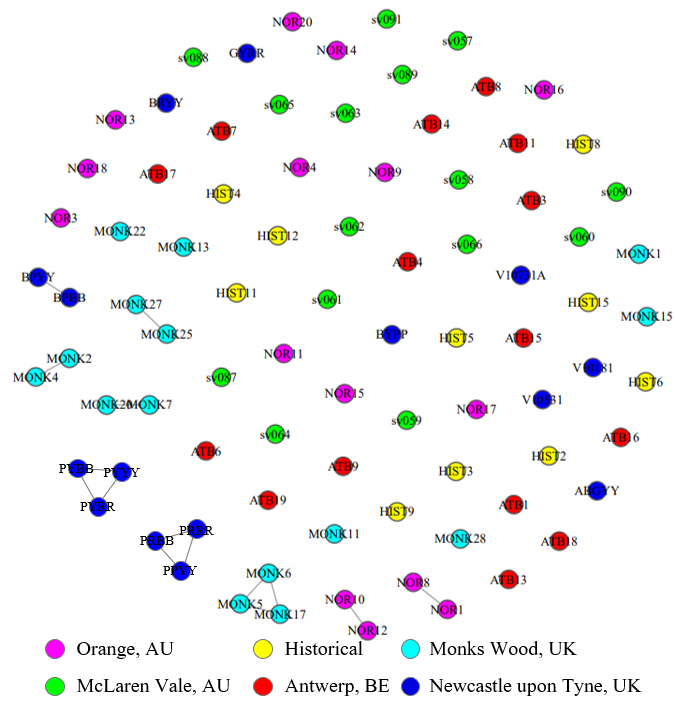
**

**Figure S1: Relatedness of *Sturnus vulgaris* contemporary and historical samples** using all SNP data, filtered, maf filter as well (HWE not excluded), with relatedness linkage displayed for individuals with a quantile of 0.004. Using the vcftools --relatedness2 flag reported these individuals above a relatedness threshold of >=0.2.


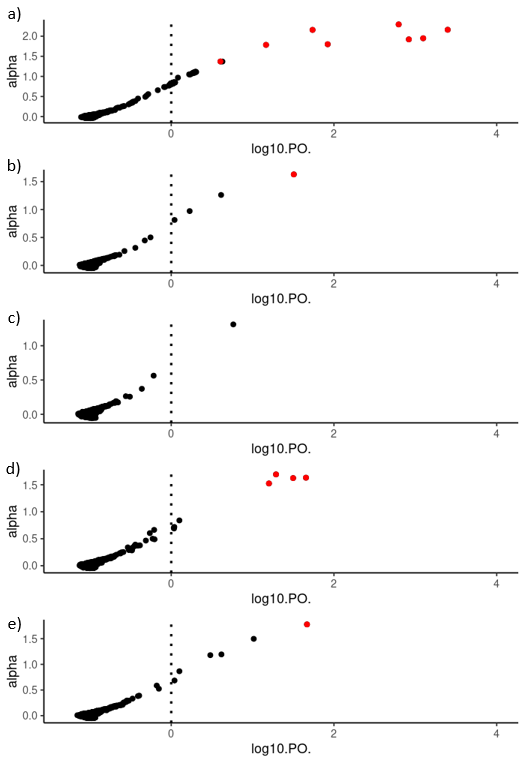


**Figure S2: *Sturnus vulgaris* outlier analysis bayescan plots** of posterior odds log likelihood for pairwise data sets of a) UK-HS b) AU_e_-HS, c) AU_s_-HS, d) UK-AU_e_, and e) UK-AU_s_, with outlier SNPs displayed in red.


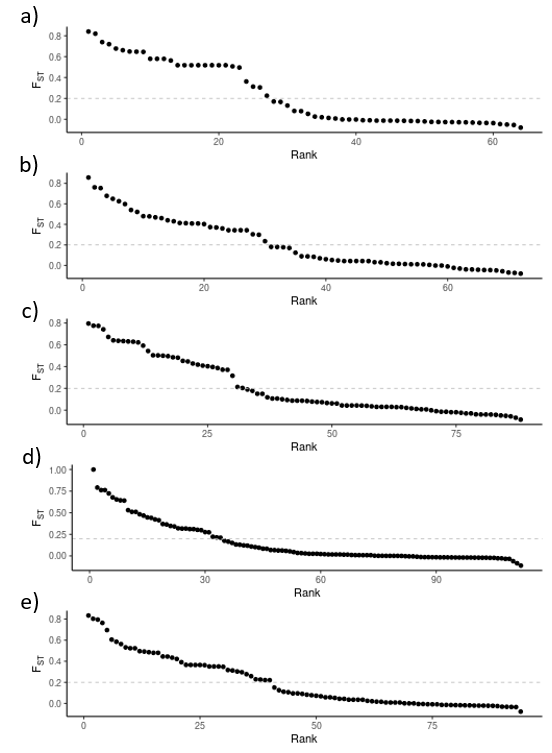


**Figure S3: *Sturnus vulgaris* outlier analysis SNP FST** **plots** for those flagged in the outlier sliding windows for pairwise data sets of a) UK-HS b) AU_e_-HS, c) AU_s_-HS, d) UK-AU_e_, and e) UK-AU_s_. SNPs above the threshold (dotted line) were retained as outliers (Fst > 0.2).


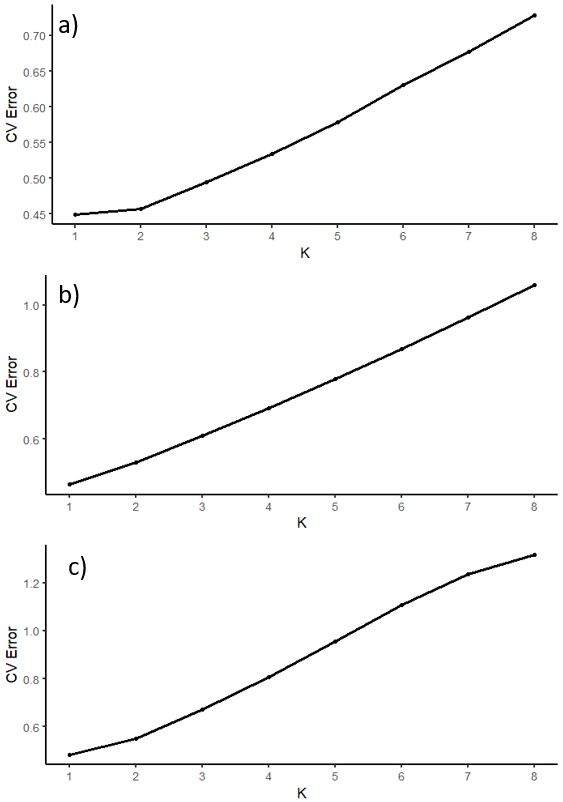


**Figure S4: *Sturnus vulgaris* admixture** **cross validation (CV) error profiles** for admixture runs for a) all samples, b) historical and contemporary native range samples, and c) contemporary invasive range samples.


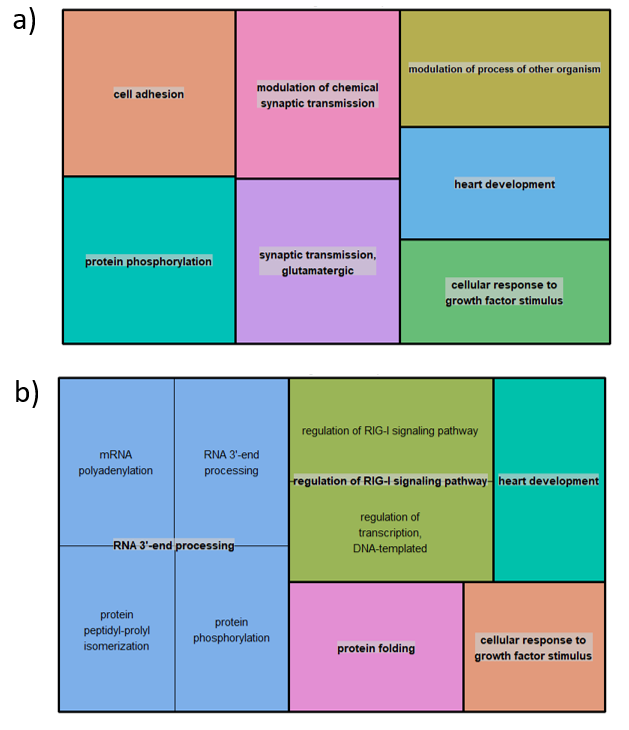


**Figure S5: REVIGO output of biological processes GO terms associated with putative genes under selection in *Sturnus vulgaris*** for SNPs reported in the a) divergent selection, and b) parallel selection data sets (colours do not match across panels).


**
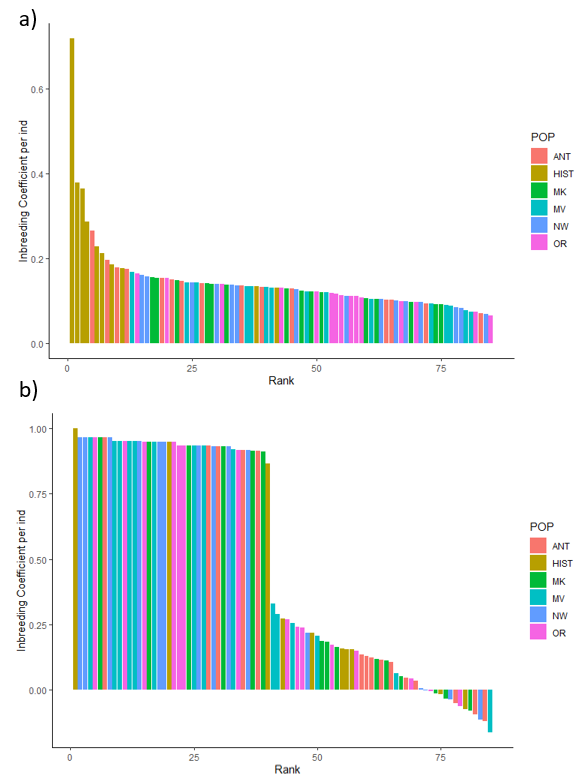
**

**Figure S6: Bioinformatics sexing of *Sturnus vulgaris*** from six sample sites with panel a) inbreeding coefficient (y-axis) across all autosomal SNPs for samples, in rank order, and panel b) inbreeding coefficient (y-axis) across all sex chromosome SNPs for samples. Females (ZW) would be expected to have high inbreeding coefficients (approaching 1) on the sex chromosome as they will have inflated homozygosity due to hemizygous sex SNPs, while males (ZZ) will have higher heterozygosity and lower inbreeding scores. We interpret individuals to the left of the ‘break point’ (sharp decrease in ranked inbreeding coefficient) as females, and the left as males. High inbreeding coefficient across autosomes in historic samples is indicative of allelic dropout due to sample quality. Inbreeding coefficients were calculated in vcftools.

**
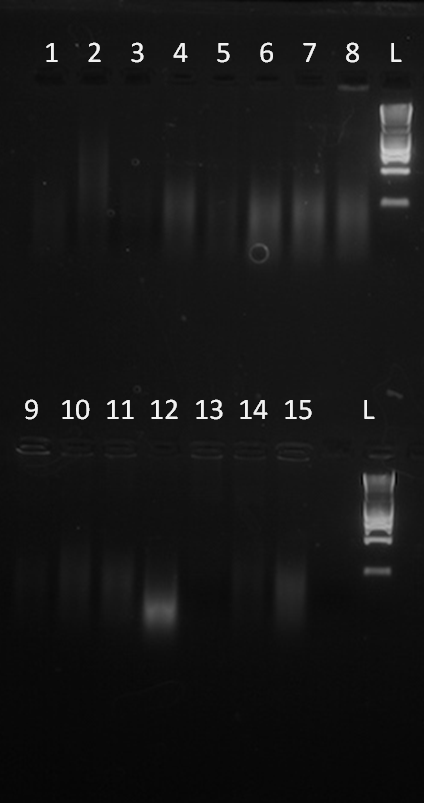
**

**Figure S7: Gel of historical *Sturnus vulgaris* samples**. 4ul of extracted DNA on 2% agarose gel. Numbers above wells denote sample ID corresponding to Table S1. L denotes HyperLadder1 (First band in HL I = 200bp, second band = 400 bp). Samples were diluted by approximately half from the concentrations reported in Table S1.


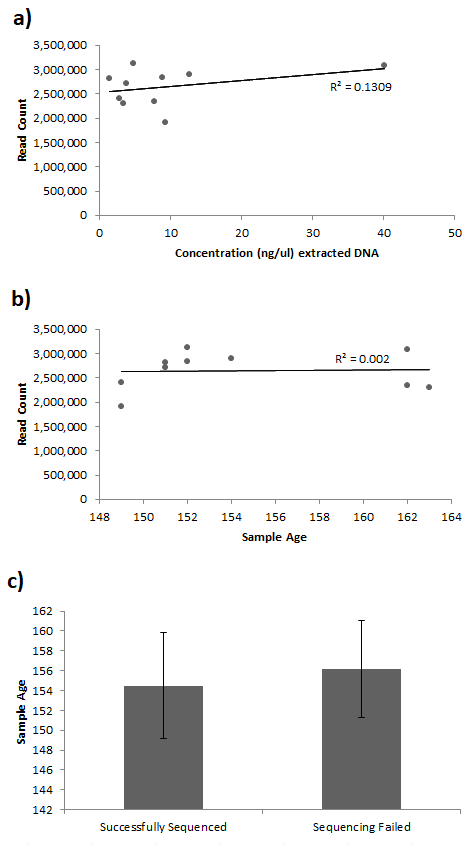


**Figure S8: Historical *Sturnus vulgaris* sample sequencing assessment** with panel a) depicting the relationship between raw read count and sample concentration, panel b) depicting the relationship between raw read count and sample age, and panel c) depicting the average sample age (+/- standard deviation) for the 10 successfully sequenced, and 5 failed historical samples.


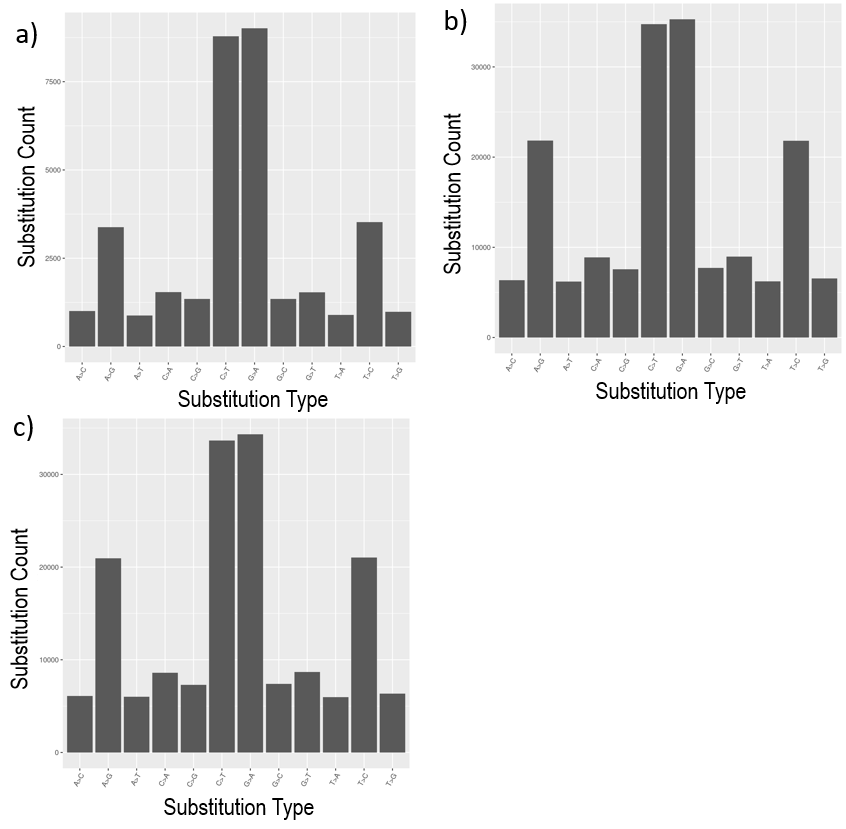


**Figure S9:** ***Sturnus vulgaris* SNP data per base substitution counts** in the unfiltered data set for a) historical, b) contemporary native range, and c) Australian *Sturnus vulgaris* samples.


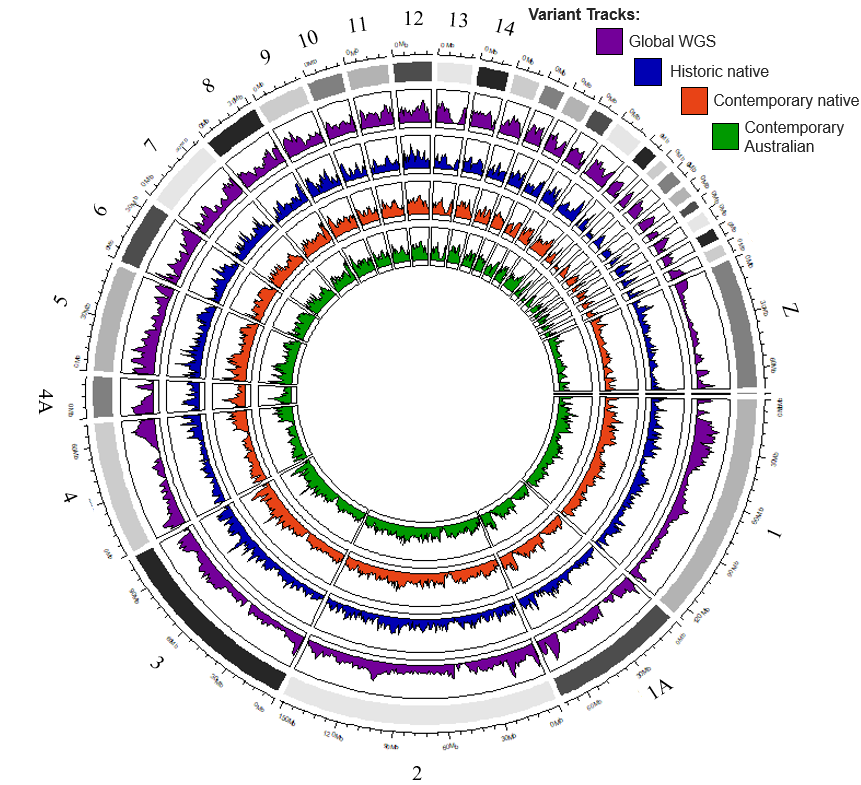


**Figure S10: *Sturnus vulgaris* WGS and reduced representation sequencing SNP variant density** plotted in 1,000,000 bp windows around the genome (first 30 scaffolds) for Illumina whole genome global starling variant data (purple), historical samples (blue), contemporary native range samples (red) and contemporary Australian samples (green).


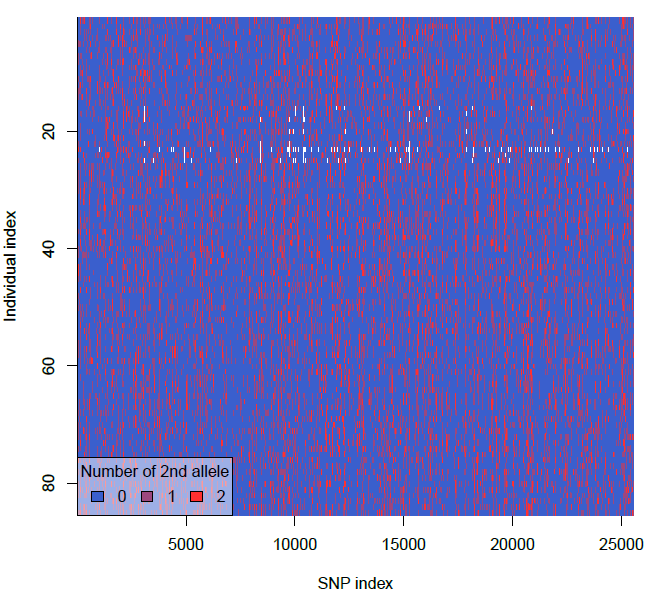


**Figure S11: Smear plot of *Sturnus vulgaris* reduced representation sequencing SNP data,** using the processed and filtered BWA-Aln variants.

**
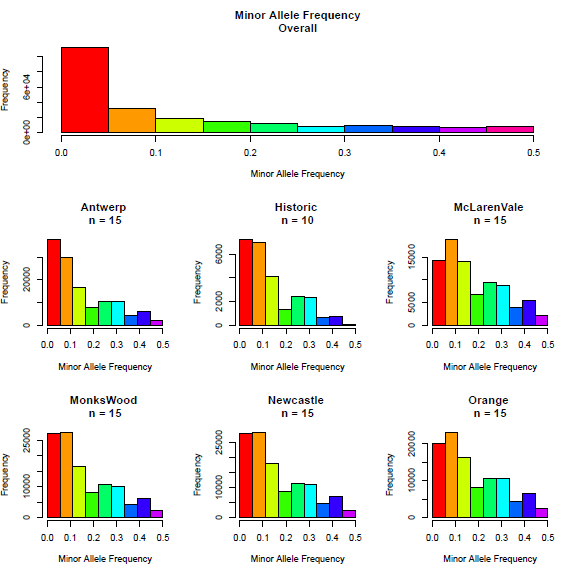
**

**Figure S12: *Sturnus vulgaris* reduced representation sequencing SNP data MAF profiles** for complete data set overall and per sample site.

**Table S1: *Sturnus vulgaris* historical native range museum samples (Tring BMNH) metadata.**

| **Sample ID** | **BMNH ID** | **Date of Collection** | **Conc. (ng/ul) extracted DNA** | **Successfully sequenced** | **Reads Count** |
| --- | --- | --- | --- | --- | --- |
| 1 | 87.7.1.2 | 01/1868 | 2.49 | NO | - |
| 2 | 2016.14.2 | 20/05/1869 | 3.82 | YES | 2,705,845 |
| 3 | 87.7.1.3 | 27/12/1869 | 1.40 | YES | 2,809,003 |
| 4 | 88.9.20.3 | 03/09/1868 | 4.78 | YES | 3,122,538 |
| 5 | 87.7.1.4 | 01/2/1871 | 2.86 | YES | 2,397,626 |
| 6 | 72.10.3.11 | 10/1871 | 9.34 | YES | 1,906,213 |
| 7 | 89.3.6.214 | 25/10/1866 | 10.18 | NO | - |
| 8 | 1889.3.6(215) | 17/03/1866 | 12.67 | YES | 2,902,553 |
| 9 | 79.4.5.879 | 05/1857 | 3.40 | YES | 2,295,671 |
| 10 | 97.11.10.729 | 22/06/1869 | 5.94 | NO | - |
| 11 | 81.5.1.3222 | 12/05/1858 | 7.81 | YES | 2,349,968 |
| 12 | 81.5.1.3227 | 05/06/1858 | 40.20 | YES | 3,076,038 |
| 13 | 81.5.1.3231 | 23/02/1857 | 1.72 | NO | - |
| 14 | 81.5.1.3232 | 31/1/1859 | 4.16 | NO | - |
| 15 | 81.5.3233 | 04/1868 | 8.85 | YES | 2,843,194 |

**Table S2: Mapping reads of historical and contemporary *Sturnus vulgaris*** using three different mapping pipelines; BWA-aln, BWA-mem, and Bowtie2-GATK. Mapped reads percentages were identified using the samtools flagstat function. Loci and variant counts are as reported by either Stacks *populations* for the bwa mapping, or by samtools for the gatk mapping. Individual missingness was calculated by using the vcftools --missing-indv flag.

|  |  | **Raw Data** | **Unfiltered data set** |  | **Filtered data set** |  |
| --- | --- | --- | --- | --- | --- | --- |
|  |  | **Mapped reads %** | **No. of loci/variant sites** | **Average Ind Data Missing (%)** | **No. of variant sites** | **Average Ind Data Missing (%)** |
| **BWAAln-Stacks** | **Contemporary & Historical** | 57.63 | 411,298/239,538 | 35.85 (historic: 83.28) | 13,722 | 10.08 (historic: 80.43) |
|  | **Historical** | 8.53 | 250,463/30,662 | 35.07 | 2,744 | 5.00 |
| **BWAMem-stacks** | **Contemporary & Historical** | 85.36 | 412,079/243,589 | 34.76 (historic: 81.57) | 14,929 | 9.61 (historic: 76.63) |
|  | **Historical** | 53.95 | 263,686/34,832 | 33.05 | 4,331 | 4.13 |
| **Bowtie2-GATK** | **Contemporary & Historical** | 89.17 | 5879/5015 | 60.25 (historic:  74.58) | 715 | 28.61 (51.03) |
|  | **Historical** | 65.24 | 1235/791 | 57.51 | 500 | 25.08 |

**Table S3: *Sturnus vulgaris* SNPs discovered by each outlier identification method.** Diagonal values represent the total number of SNPs flagged that were unique to each method. Pairwise overlap between outlier identification methods are represented in the cells below the diagonal. Total number of SNPs flagged in each analysis indicated in brackets. Columns and rows will not necessarily sum to the total number of SNPs within each method, as the same SNP may be flagged in multiple pairwise comparisons (for AU-HS and UK-AU, as outliers across AU_east_ and AU_south_ are also compared).

| **UK-HS** |  |  | |  |  |
| --- | --- | --- | --- | --- | --- |
|  |  | **UK-HS** | |  |  |
|  | **METHOD** | **Bayescan (8)** | **Windows 0.99 (27)** |  |  |
| **UK-HS** | **Bayescan (8)** | 4 | - |  |  |
|  | **Windows 0.99 (27)** | 4 | 23 |  |  |
| **AU-HS** |  |  | |  | |
|  |  | **AUe-HS** | | **AUs-HS** | |
|  | **METHOD** | **Bayescan (1)** | **Windows 0.99 (30)** | **Bayescan (0)** | **Windows 0.99 (32)** |
| **AUe-HS** | **Bayescan (1)** | 0 | - | - | - |
|  | **Windows 0.99 (30)** | 1 | 20 | - | - |
| **AUs-HS** | **Bayescan (0)** | 0 | 0 | 0 | - |
|  | **Windows 0.99 (32)** | 1 | 10 | 0 | 22 |
| **UK-AU** |  |  |  |  |  |
|  |  | **UK-AUe** | | **UK-AUs** | |
|  | **METHOD** | **Bayescan (4)** | **Windows 0.99 (34)** | **Bayescan (1)** | **Windows 0.99 (40)** |
| **UK-AUe** | **Bayescan (4)** | 1 | - | - | - |
|  | **Windows 0.99 (34)** | 3 | 28 | - | - |
| **UK-AUs** | **Bayescan (1)** | 0 | 1 | 0 | - |
|  | **Windows 0.99 (40)** | 1 | 4 | 1 | 36 |

**Table S4:** **Summary of allelic frequency** **for outlier SNPs in *Sturnus vulgaris*** under divergent and parallel selection as calculated by Stacks. * asterisk indicates variant appearing in both lists.

| **CLASSIFY** | **CHROM** | **POS** | **Mapped Gene/Genes** | **Major Allele** | **UK Major freq** | **AU_east_ Major freq** | **AU_south_ Major freq** | **HIST Major freq** |
| --- | --- | --- | --- | --- | --- | --- | --- | --- |
| Divergent | chromosome1 | 111389366 |  | C | 1 | 0.4 | 1 | 1 |
| Divergent | chromosome2 | 43646670* | TGFBR2 | T | 0.452381 | 0.454545 | 1 | 1 |
| Divergent | chromosome2 | 125529523 |  | A | 0.965517 | 0.785714 | 0.464286 | 1 |
| Divergent | chromosome3 | 23635575 |  | G | 0.571429 | 0 | 0.25 | 0.875 |
| Divergent | chromosome3 | 24369993 | GRIK2 | G | 0.75 | 0 | 0 | 0.75 |
| Divergent | chromosome5 | 35125799 | NRXN3 | A | 0.840909 | 0.266667 | 0.625 | 1 |
| Divergent | chromosome12 | 4462195 | CACNA2D3 | C | 1 | 0.583333 | 0.357143 | 1 |
| Divergent | chromosome13 | 18573988 | ANKHD1 | G | 0.955556 | 0.766667 | 0.566667 | 0.75 |
| Divergent | chromosome15 | 10705684 |  | G | 0.833333 | 1 | 0.2 | 1 |
| Divergent | chromosome18 | 2041249 |  | A | 1 | 0.454545 | 0.846154 | 1 |
| Divergent | chromosome24 | 5999859 |  | A | 1 | 1 | 0.5 | 0.6 |
| Divergent | chromosome28 | 4287460 | Unknown Protein | C | 0.142857 | 1 | 1 | 1 |
| Divergent | chromosomeZ | 2191887 |  | A | 0.942308 | 0.944444 | 0.4 | 1 |
| Divergent | chromosomeZ | 31262637 |  | A | 0.8 | 0.866667 | 0.233333 | 0.833333 |
| Divergent | chromosome29 | 1358881 | NATT4/DNMT1 | G | 0.9 | 0.8 | 0.4375 | 1 |
| Parallel | chromosome1 | 3428749 |  | T | 1 | 1 | 1 | 0.666667 |
| Parallel | chromosome1 | 83513325 |  | G | 0.90625 | 0.833333 | 1 | 0.357143 |
| Parallel | chromosome2 | 43646670* | TGFBR2 | T | 0.452381 | 0.454545 | 1 | 1 |
| Parallel | chromosome3 | 71978252 |  | G | 0.5 | 0.5 | 0.566667 | 1 |
| Parallel | chromosome11 | 18202987 |  | C | 1 | 1 | 1 | 0.666667 |
| Parallel | chromosome13 | 3396982 | TRIM25 | G | 1 | 1 | 1 | 0.666667 |
| Parallel | chromosome19 | 8454504 | MYBBP1A | C | 0.939394 | 0.9 | 0.928571 | 0.25 |
| Parallel | chromosomeZ | 19762165 | CWC27 | C | 1 | 1 | 1 | 0.666667 |
| Parallel | chromosomeZ | 21621921 | TENT2 | G | 0.947368 | 1 | 0.928571 | 0.285714 |
| Parallel | chromosomeZ | 24764608 |  | A | 0.966667 | 1 | 0.916667 | 0.142857 |
| Parallel | chromosomeZ | 33855811 |  | C | 1 | 1 | 1 | 0.625 |
| Parallel | chromosome29 | 1276366 | TRIM28 | T | 1 | 1 | 1 | 0.625 |

**Table S5:** **Summary of putative genes under spatial and temporal selection in *Sturnus vulgaris*** that were mapped to by the outlier SNPs reported at putatively divergent or parallel. Definitions pulled from GeneCards (Stelzer *et al.* 2016). * asterisk indicates variant appearing in both lists.

| **Gene** | **Name** | **Biological Function** | |
| --- | --- | --- | --- |
| **Divergent Genes** | |  | |
| *TGFBR2** | *Transforming Growth Factor Beta Receptor 2* | The protein encoded by this gene is a transmembrane protein that has a protein kinase domain, forms a heterodimeric complex with TGF-beta receptor type-1, and binds TGF-beta. This receptor/ligand complex phosphorylates proteins, which then enter the nucleus and regulate the transcription of genes related to cell proliferation, cell cycle arrest, wound healing, immunosuppression, and tumorigenesis. | |
| *GRIK2* | *Glutamate Ionotropic Receptor Kainate Type Subunit 2* | Glutamate receptors are the predominant excitatory neurotransmitter receptors in the mammalian brain and are activated in a variety of normal neurophysiologic processes. Mutations in this gene have been associated with autosomal recessive cognitive disability. | |
| *NRXN3* | *Neurexin-3* | This gene encodes a member of a family of proteins that function in the nervous system as receptors and cell adhesion molecules. | |
| *CACNA2D3* | *Voltage-dependent calcium channel subunit alpha-2/delta-3* | The alpha-2/delta subunit of voltage-dependent calcium channels regulates calcium current density and activation/inactivation kinetics of the calcium channel. | |
| *ANKHD1* | *Ankyrin repeat and KH domain-containing protein 1* | May play a role as a scaffolding protein that may be associated with the abnormal phenotype of leukemia cells. Isoform 2 may possess an antiapoptotic effect and protect cells during normal cell survival through its regulation of caspases. | |
| *NATT4* | *Natterin-4* | First discovered as a toxin in fish, shows nociceptive, edema-inducing and kininogenase activity with release of kallidin from low molecular weight kininogen. |  |
| *DNMT1* | *DNA Methyltransferase 1* | This gene encodes an enzyme that transfers methyl groups to cytosine nucleotides of genomic DNA. This protein is the major enzyme responsible for maintaining methylation patterns following DNA replication and shows a preference for hemi-methylated DNA. |  |
| **Parallel Genes** | |  | |
| *TGFBR2** | *Transforming Growth Factor Beta Receptor 2* | The protein encoded by this gene is a transmembrane protein that has a protein kinase domain, forms a heterodimeric complex with TGF-beta receptor type-1, and binds TGF-beta. This receptor/ligand complex phosphorylates proteins, which then enter the nucleus and regulate the transcription of genes related to cell proliferation, cell cycle arrest, wound healing, immunosuppression, and tumorigenesis. |  |
| *TRIM25* | *E3 ubiquitin/ISG15 ligase TRIM25* | The protein encoded by this gene is a member of the tripartite motif (TRIM) family. The TRIM motif includes three zinc-binding domains, a RING, a B-box type 1 and a B-box type 2, and a coiled-coil region. The protein localizes to the cytoplasm. The presence of potential DNA-binding and dimerization-transactivation domains suggests that this protein may act as a transcription factor, similar to several other members of the TRIM family. Expression of the gene is upregulated in response to estrogen. | |
| *MYBBP1A* | *Myb-binding protein 1A-like protein* | This gene encodes a nucleolar transcriptional regulator that was first identified by its ability to bind specifically to the Myb proto-oncogene protein. The encoded protein is thought to play a role in many cellular processes including response to nucleolar stress, tumor suppression and synthesis of ribosomal DNA. | |
| *CWC27* | *CWC27 spliceosome associated cyclophilin* | Gene Ontology (GO) annotations related to this gene include peptidyl-prolyl cis-trans isomerase activity. | |
| *TENT2* | *Poly(A) RNA polymerase GLD2* | Cytoplasmic poly(A) RNA polymerase that adds successive AMP monomers to the 3'-end of specific RNAs, forming a poly(A) tail. | |
| *TRIM28* | *Tripartite Motif Containing 28* | The protein encoded by this gene mediates transcriptional control by interaction with the Kruppel-associated box repression domain found in many transcription factors. The protein localizes to the nucleus and is thought to associate with specific chromatin regions. | |

**Table S6: Chi-square table for the test of statistical association between SNPs categorised as under selection in *Sturnus vulgaris* versus the chromosome type** they reside in for *Sturnus vulgaris* DArT-Seq (rounded to 1dp).

|  |  |  | **Chromosome Category** | | |  |
| --- | --- | --- | --- | --- | --- | --- |
|  |  |  | **Marco** | **Micro** | **Sex-** | **Total** |
| **SNP category** | **Divergent SNPs** | Count Exp. Count Residual | 7 11.3  -1.3 | 6  2.8  1.9 | 2  0.9  1.2 | 15  15 |
|  | **Parallel SNPs** | Count Exp. Count Residual | 4  8.3  -1.5 | 3  2.0  0.7 | 4  0.6  4.2 | 11  11 |
|  | **Putative SNPs under selection** | Count Exp. Count Residual | 59  64.3  -0.7 | 16  15.8  0.1 | 10  4.9  2.3 | 85  85 |
|  | **No Selection** | Count Exp. Count Residual | 3763  3749.0  0.2 | 916  920.4  -0.1 | 278  287.6  -0.6 | 4957  4957 |
| **Total** |  | **Count** | 3833 | 941 | 294 | 5068 |
|  |  | **Expected Count** | 3833 | 941 | 294 | 5068 |
